# Food additives containing potassium, phosphorus, and sodium in ultra-processed foods: potential harms to individuals with chronic kidney disease

**DOI:** 10.1038/s41430-025-01600-6

**Published:** 2025-03-21

**Authors:** Valeria Cecchini, Alice Sabatino, Barbara Contzen, Carla Maria Avesani

**Affiliations:** 1 Division of Renal Medicine, Baxter Novum. Department of Clinical Science, Intervention and Technology. Karolinska Institutet, Solna, Sweden; 2 Nephrologik, Nutritional therapy for patients with chronic kidney disease, Bergisch Gladbach, Germany

**Keywords:** Nutrition, Kidney diseases, Education

## Introduction

In recent decades, global dietary patterns have undergone profound changes. The food landscape is marked by unprecedented shifts in food availability and consumer demand for accessible, convenient, and appetising food options [1]. This evolution has been driven by the combination of sophisticated food science technologies and the modernisation of grocery and retailing practices over the past seven decades, resulting in an increase of ultra-processed foods (UPFs) throughout global food chains [1, 2]. Subsequently, the globalisation process has further disseminated these products worldwide [3].

The rise of UPFs plays a central role in the ongoing nutrition transition, particularly impacting low- and middle-income countries [3]. This transition is characterised by a divergence from traditional local dietary habits towards a Western diet, represented by a high prevalence of calorie-dense foods containing elevated amounts of sugars, or non-caloric artificial sweeteners, saturated fat, and refined grains [3]. These characteristics, alongside sophisticated marketing strategies and the incorporation of cosmetic additives, are foundational to UPFs’ wide dispersion [1].

The consumption of UPFs varies greatly around the globe with lower consumption in the Mediterranean countries (10–20% of the total energy intake) and higher consumption in the United States of America, Canada, Australia, United Kingdom, and Sweden (45–60% of the total energy intake) [3, 4]. Increased consumption of UPFs is associated with a range of adverse health outcomes, such as type 2 diabetes, cardiovascular disease (CVD), obesity, cancer, chronic kidney disease (CKD), and mental health disorders [5, 6].

In the context of CKD, high UPF consumption can have different outcomes, it can contribute to the development of the disease [7] and, for individuals who have been diagnosed with CKD, it can potentially further worsen the metabolic derangements that appear as kidney function decreases [6]. In particular, elevated consumption of UPFs is associated with an increased risk of all-cause mortality and kidney function decline in renal transplant patients [8].

The 2024 *KDIGO Clinical Practice Guideline for the evaluation and management of CKD* recommends that individuals with CKD adopt a varied diet that prioritises plant-based foods over those of animal origin. It also advises reducing the intake of UPFs [9]. Emphasising plant-based diets for patients with CKD not on dialysis offers several benefits, including reduced bioavailability of plant phosphorus due to its binding to phytate, increased dietary fibre supporting gut microbiota health and reducing uraemic toxins, and plant-derived anions potentially mitigating metabolic acidosis [10, 11].

Limiting UPFs for patients with CKD may be particularly beneficial, as they often contain added sugar, salt, hydrogenated fat and additives containing potassium, phosphorus, and sodium in their molecular structure. More specifically, consuming additives containing potassium and phosphorus may be associated with a higher risk of hyperkalemia and hyperphosphatemia, respectively [6, 12]. Additionally, phosphorus additives, often emulsifiers, have been associated with gut dysbiosis [13], whereas sodium additives may be linked to difficulties in managing blood pressure [12].

Considering this, the current review aims to increase awareness on what UPFs are, their consumption, and the potential harmful effects of food additives to individuals with CKD, ultimately empowering them to make informed dietary choices. Additionally, we offer practical guidance to healthcare practitioners on navigating the current dietary scene for patients with CKD stages 3–5, non-dialysis, and dialysis, concerning the intake of UPFs. Ultimately, this review aims to foster effective approaches to the treatment of CKD stages 3–5 by choosing foods wisely.

## Identifying UPFs with the NOVA food classification system

The NOVA food classification system was coined by the researchers at the Centre for Epidemiological Studies in Health and Nutrition at the School of Public Health, University of São Paulo, Brazil [14] and has since then been recognised as the most specific and practical approach to identify UPFs [2]. In this classification, food and beverages are divided into four categories: unprocessed and minimally processed foods (Group 1: fruit and vegetables, eggs, fish, meat, poultry, legumes, etc.), processed culinary ingredients (Group 2: salt, sugar, honey, vegetable oils, etc.), processed foods (Group 3: canned and bottled vegetables, legumes in brine, breads, cakes, cheeses, made only from Group 1 and 2 foods), and UPFs (Group 4: soft drinks, luncheon meats, hot dogs, instant noodles, ready to heat/eat products, etc.) based on the nature, extent, and purpose of the industrial processes that they undergo [14]. These categories are described in more detail in Supplementary Table 1. UPFs are defined as formulations that do not include whole food, but instead use ingredients such as modified starches, protein isolates, partially hydrogenated fats and others, that are assembled by sophisticated industrial procedures, employing heavy machinery for ingredient combination [15]. These methodologies often entail extrusion, moulding, pre-frying, and the inclusion of cosmetic additives, such as colours, flavour enhancers, emulsifiers, carbonating, anti-foaming, and glazing agents for reinstating the sensory characteristics (e.g. texture and flavour) and the original appearance (e.g. colour) in order to increase the products’ palatability and sales [15]. Of note, the European Union (EU) regulations allow the use of colours to reinstate the appearance lost during the industrial processes [16], and this constitutes an example of the concept of cosmetic additives, as delineated by the NOVA food classification system.

## The role of UPFs in health and disease development

The global increase in the consumption of UPFs has been associated with adverse health outcomes and the development of non-communicable diseases [4, 5]. The mechanisms behind these associations are yet in their infant stages. Still, it is believed that the dietary shift from whole foods—such as fruits, vegetables, legumes, whole grains, and nuts, which are rich in vitamins, antioxidants, and fibres—to UPFs plays a significant role. UPFs are usually energy-dense and typically contain excessive amounts of hydrogenated fat, sugar, sodium, and food additives. The latter have been highly implicated in driving chronic inflammatory diseases, with particularly adverse effects to the gut microbiota [13]. This is due to the by-products coming from the combination of food additives and newly-formed compounds such as acrolein, advanced glycation end-products (AGEs), furans, acrylamide, and polycyclic aromatic hydrocarbons [17]. In some chronic disease contexts, additives can have further adverse effects. For individuals with diabetes, ingredients like maltose, high-fructose corn syrup, and other sucrose replacements can have a negative impact on glycemic control, especially if individuals are not aware of such ingredients [18]. However, recent evidence has highlighted that certain subgroups of UPFs, such as dark, whole grain bread and cereals, and some yoghurts are associated with a lower risk of type 2 diabetes [18]. Therefore, it can be argued that, although these products may be classified as UPFs, they could also be part of a healthy dietary pattern in accordance with dietary guidelines, which may mitigate the risk of developing type 2 diabetes. Additionally, these products do not exhibit the same quasi-addictive properties as other UPFs, thereby emphasising their distinct role within the diet. When it comes to CKD as health outcome, a convincing body of literature, summarised in a systemic review and meta-analysis, has consistently shown that in the general population, individuals who exhibit a higher intake of UPFs have higher chances to develop CKD over time [7]. Of importance is to understand the role that food additives may play in CKD and their potential contribution to further worsening metabolic derangements inherent to the disease, such as hyperkalaemia and hyperphosphatemia.

## Food additives: definition and their relation to health in individuals with CKD

Food additives are substances utilised for several purposes, such as preservation, colouring, flavour enhancement, and texture improvement. They are described as substances that are not typically consumed on their own or commonly used as culinary ingredients, irrespective of their nutritional content [16]. Food additives can be found in both processed foods and UPFs, but they are more abundant in UPFs. While the additives in processed foods are used to preserve their microbiological profile, cosmetic additives are typically absent.

According to the EU legislation, they are permitted for 26 specific technological purposes [19]. When added to food during production, processing, or packaging, additives become integral food constituents that should be taken into consideration [19]. Within the additives’ domain, there are several chemical formulations, and their distinct structures serve different purposes, as described in Table 1 [20].

**Table 1:** Number of authorised food additives containing potassium, phosphorus, and sodium in the European Union, their purposes and potential harmful effects to kidney health.

Mineral	N of authorised additives	Food categories	Purposes	Potential harmful effects
Potassium	41			
	**Group I**^a^: E 261, E 326, E 332, E 336, E 337, E 351, E 402, E 407, E 407a, E 415, E 418, E 440, E 470a, E 472c, E 501, E 508, E515, E 525, and E 577	Potassium-containing additives are authorised in food categories 1, 2, 3, 4, 5, 6, 7, 8, 9, 10, 11, 12, 13, 14, 15, 16, 17, and 18	Preservation, antioxidation, emulsification, stabilisation, thickening, gelling, acidity regulators, leavening, sodium replacement, flavour enhancer, colour stabiliser, and sweetening	These additives can exacerbate hyperkalemia
	**Other additives that may be regulated combined**: E 202, E 212, E 224, E 228, E 249, E 252, E 283, E 340, E 357, E 522, E 555, E 622, E 628, and E 632			
Phosphorus	44			
	**Group I**: E 322, E 1200, E 1410, E 1412, E 1413, E 1414, and E 1442 **Group II**^b^: E 101	Phosphorus-containing additives are authorised in food categories 1, 2, 3, 4, 5, 6, 7, 8, 9, 10, 11, 12, 13, 14, 15, 16, 17, and 18	Preservation, emulsification, stabilisation, thickening, gelling, antioxidation, acidity regulators, leavening, and colouring	These additives can exacerbate hyperphosphatemia and bone-mineral diseases
	**Other additives that may be regulated combined**: E 338, E 339, E 340, E 341, E 343, E 450, E 451, and E 452, E 626, E 627, E 628, E 630, E 631, E 632, E 633, E 634, and E 635			
Sodium	88			
	**Group I**: E 262, E 301, E 325, E 331, E 335, E 337, E 350, E 401, E 407, E 407a, E 415, E 418, E 440, E 466, E 469, E 470a, E 472c, E 500, E 514, E 524, E 576, E 640, E 1404, E 1450, E 1451 **Group II**: E 101 **Group III**^c^: E 104, E 110, E 122, E 124, E 129, E 131, E 132, E 133, E 142, E 151, E 155	Sodium-containing additives are authorised in food categories 1, 2, 3, 4, 5, 6, 7, 8, 9, 10, 11, 12, 13, 14, 15, 16, 17, and 18	Preservation, antioxidation, emulsification, stabilisation, thickening, gelling, leavening, acidity regulator, anti-caking, colour stabiliser, and flavour enhancer	These additives can increase blood pressure, renal plasma flow, and glomerular filtration pressure
	**Other additives that may be regulated combined**: E 211, E 215, E 219, E 221, E 222, E 223, E 250, E 251, E 281, E 339, E 356, E 481, E 521, E 554, E 621, E627, E 631, and E 635			

Food categories: 1 Dairy products and analogues; 2 Fats and oils and fat and oil emulsions; 3 Edible ices; 4 Fruit and vegetables; 5 Confectionery; 6 Cereals and cereal products; 7 Bakery wares; 8 Meat; 9 Fish and fisheries products; 10 Egg and egg products; 11 Sugars, syrups, honey and table-top sweeteners; 12 Salts, spices, soups, sauces, salads and protein products; 13 Foods intended for particular nutritional uses as defined by Directive 2009/39/EC; 14 Beverages; 15 Ready-to-eat savouries and snacks; 16 Desserts excluding products covered in categories 1, 3 and 4; 17 Food supplements as defined in Directive 2002/46/EC excluding food supplements for infants and young children; 18 Processed foods not covered by categories 1 to 17, excluding foods for infants and young children.

^a^Group I: no maximum numerical limit is established. However, these substances must be utilised following good manufacturing practices. Their usage should be limited to what is essential to fulfil their intended purpose, guaranteeing that the consumers are not deceived.

^b^Group II: food colours allowed at *Quantum satis*.

^c^Group III: food colours with combined maximum limit.

Current research has identified food products that contain potassium-, phosphorus-, and sodium-based additives and quantified their proportions within various additives [21–23]. However, the exact amount of additives in foods remains unknown, as the assessment of the foods’ nutritional composition does not quantify how much of the mineral comes from an organic or an inorganic source, making it difficult to differentiate between naturally occurring minerals and those added as additives in processed foods and in UPFs. Without accurate knowledge on the amount of additives containing potassium, phosphorus, and sodium added to foods, it becomes difficult to develop clear dietary counselling regarding processed foods and UPFs when hyperkalemia and hyperphosphatemia are present in patients with CKD stages 3 to 5. Therefore, up to now, dietary counselling assumes that the potassium, phosphorus and sodium content in UPFs primarily comes from additives, and thus, that these mineral sources may be more harmful than their naturally occurring counterparts for patients with CKD and concomitant hyperkalaemia and/ or hyperphosphatemia.

### Potassium additives

Considering the lack of information on additives present in food, the findings of Martínez-Pineda et al. [23] are relevant, as the authors assessed the prevalence of potassium-containing additives, even though they did not quantify the amount of these additives within the food.

Their study identified 41 approved additives containing potassium within the EU. Additionally, they found that 19% of these additives had a potassium content exceeding 40%, with half of this 19% classified under Group I (indicates additives permitted at *Quantum satis*, without a maximum quantitative limit).

After a comprehensive review of the current legislation regarding additives containing phosphorus and sodium according to the Commission Regulation (EU) No. 1129/2011 [16, 24] we identified 44 phosphorus- and 88 sodium-containing additives, which are authorised for numerous purposes (Table 1).

### Phosphorus additives

An extensive analysis examining the molecular structure, weight, and purity of phosphorus additives, including market specifics, as outlined in the EU Regulation no. 231/2012 [20], identified that 30% of the additives containing phosphorus in their formulation contain a moderate amount of phosphorus, with none surpassing 31.6% of phosphorus content (Supplementary Table 2). However, it is worth acknowledging that the analysis omitted commonly used phosphorus additives, such as polyphosphates (E 452), due to their complex and mutable chemical compositions [21]. All additives classified under the medium content category, except E 541, are permitted across all food categories at a maximum level of 10,000 mg/kg or L, with restrictions applicable solely to specific categories [24]. These additives serve numerous technological functions in food, including acidity regulation, melting salts, leavening, emulsification, and stabilisation, making them a common, yet hidden source of phosphorus in a broad variety of foods [23]. Despite the established acceptable daily intake (ADI) for phosphates being 40 mg/kg of body weight for the general population [25], individuals with CKD are more vulnerable to these additives, underscoring the need for comprehensive food labelling practices to precisely quantify phosphorus content in packaged food.

### Sodium additives

We identified 88 sodium-containing food additives approved within the EU. Findings revealed that 17% of these additives contain a moderate amount of sodium, while 4% contain a high amount (≥40% of sodium relative to the total molecular weight). Among these, sodium carbonates (E 500) and sodium hydroxide (E 524) are compounds with sodium content exceeding 40%, both falling under Group I, as shown in Table 1. Their primary technological function is to regulate acidity in a wide variety of food items. Specifically, E 500 can be found in dehydrated milk, cheese, butter, chocolate products, sweeteners, and salt substitutes. Conversely, E 524 can be found in jams, fruit and vegetable spreads. Both additives may be found in chocolate products, processed cereal-based foods, and baby food [24]. At present, it is not possible to discern between the naturally occurring sodium and the added sodium in additive form when reading food labels. Nevertheless, hidden sodium sources in the diet of individuals with CKD stages 3 to 5 can complicate efforts to manage blood pressure and fluid overload, the latter particularly in patients in more advanced stages of CKD, potentially impacting medication effectiveness and dialysis treatment.

## Helping patients with CKD stage 3 to 5 to reduce their intake of food additives

**Fig. 1:**
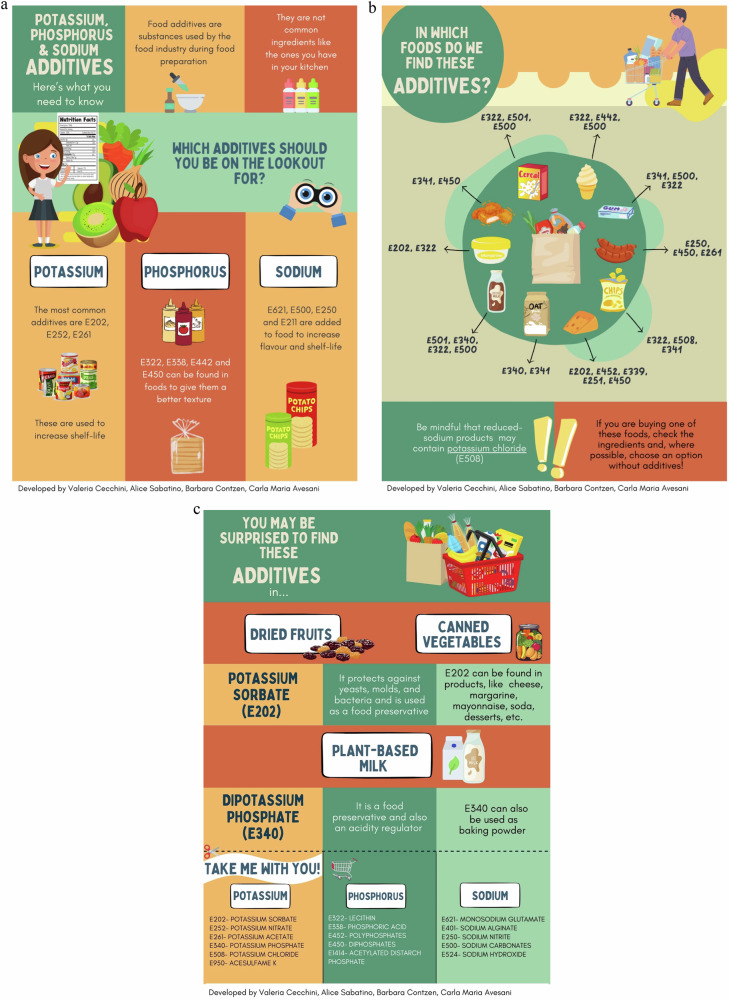
Educative material regarding food additives for patients with chronic kidney disease. **a** Food additives containing potassium, phosphorus, and sodium. **b** Sources of food additives containing potassium, phosphorus, and sodium. **c** Unexpected sources of potassium, phosphorus, and sodium additives.

Reducing the intake of food containing additives holds significant benefits for patients managing CKD stages 3–5. Empowering them with practical strategies can significantly aid their dietary choices. For patients, education begins with reading the food labels and teaching them how to carefully examine ingredient lists for additives such as potassium, phosphorus, and sodium ones. On this regard, Fig. 1A–C illustrates examples of educational leaflets designed for distribution to patients. Emphasising the preference for whole foods over UPF options can ensure enhanced nutritional quality devoid of additives, although some Group 1 foods might be high in potassium and/or phosphorus, as natural sources. Moreover, limiting the consumption of packaged foods and choosing instead fresh or frozen raw produce further minimises additive intake. Cooking at home grants individuals control over ingredient selection, facilitating the avoidance of high-additive ready-made dishes and condiments.

**Fig. 2:**
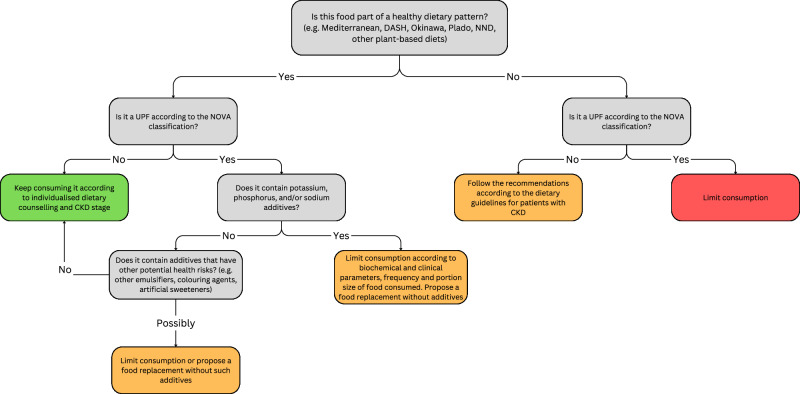
Decision tree diagram for healthcare professionals providing guidance on UPF consumption to patients with CKD. For more details regarding management of potassium and phosphorus in CKD [9, 11]. UPF ultra-processed food, CKD chronic kidney disease, DASH Dietary Approaches to Stop Hypertension, Plado Plant-dominant Low Protein Diet, NND New Nordic Diet.

Considering the available evidence showing that some groups of UPFs can be part of a healthy dietary pattern, here we propose a diagram to assist dietary counselling by dietitians, nutritionists, or other healthcare providers in educating patients about UPF consumption (Fig. 2). Finally, strategies employing a multidisciplinary approach (including social workers dedicated to enhancing access to nutritious and affordable food) are likely to empower patients in making informed food choices and reduce feelings of culpability when healthier alternatives are inaccessible.

## Conclusions

Providing guidance to individuals with CKD stages 3–5 for limiting potassium and phosphorus intake faces numerous challenges due to the underestimated and unquantified presence of additives in foods, especially UPFs. Understanding the proportion by weight of potassium, phosphorus, and sodium present in the additives does not aid in determining the overall quantity added to the final product. Therefore, effective control of these minerals may be achieved through regulatory measures at the EU level. For instance, implementation of mandatory labelling, detailing the nutritional facts and providing the total amount of potassium and phosphorus additives, would facilitate the identification of alternative options with lower content. This in turn, would allow for better control of overall potassium, phosphorus, and sodium intake, which is crucial for managing CKD. Furthermore, future studies should prioritise understanding the potential harmful doses of these additives, identifying which types are more detrimental to people’s health, and determining whether adverse health outcomes are caused solely by the additives or by the overall nutritional quality of the products.

## Supplementary information

Supplementary Table 1 and 2
