# Supplementary material for: Food additives containing potassium, phosphorus, and sodium in ultra-processed foods: potential harms to individuals with chronic kidney disease
Source: Eur J Clin Nutr. 2025 Mar 21;80(1):1–6. doi: 10.1038/s41430-025-01600-6 (PMC12783045; doi:10.1038/s41430-025-01600-6)
Supplement: Supplementary file 1 — Supplementary Table 1 and 2 [file 41430_2025_1600_MOESM1_ESM.docx]

Supplementary material

Supplementary Table 1: The NOVA food classification system and its four categories based on the nature, extent, and purpose of industrial processing (14).

Supplementary Table 2: Authorised phosphorus and sodium additives classified according to their phosphorus and sodium content, relative to the total molecular weight.

Supplementary Table 1. The NOVA food classification system and its four categories based on the nature, extent and purpose of industrial processing (14).

| **Nova food categories** | **Definition** | **Examples** |
| --- | --- | --- |
| Unprocessed and minimally processed foods (Group 1) | Unprocessed foods: edible components of plants, animals, fungi, algae once removed from nature.  Minimally processed foods: unprocessed foods with minimal industrial refinement, such as drying, freezing, or roasting. These foods maintain their natural structure and do not contain added substances or additives | Fruits, vegetables, grains, legumes, starchy roots, tubers, and fungi. Meat, eggs, fish, pasteurised milk. Pasta, couscous, and polenta from flours. Spices, herbs, teas, and coffee |
|  |  |  |
| Processed culinary ingredients (Group 2) | Substances originating from Group 1 foods or from natural sources via methods like extracting and refining. These methods are employed to create seasonings and cooking ingredients for Group 1 foods. Additives are seldom present in this category | Vegetable oils and animal fats, sugar and molasses vinegar, starches, and salt |
|  |  |  |
| Processed foods (Group 3) | Industrial food products made by incorporating one or more Group 2 ingredients to Group 1 foods using methods like canning, boiling, or baking. The principal aim of processing is to improve the foods’ durability. No additives with cosmetic functions are included in this category | Canned vegetables, legumes in brine, and salted nuts. Breads and cheese made solely by the combination of Group 1 and Group 2 foods |
| UPFs (Group 4) | Industrially modified products made up of different components, frequently lacking culinary use. Manufacturing methods of UPFs include extrusion and pre-frying. Additives with cosmetic functions, such as flavour enhancers and emulsifiers are often found in this category | Soft drinks, fruit juices from concentrate, flavoured milks and yoghurts. Packaged snacks, luncheon meats, ready-to-eat foods,  plant-based alternatives |

Supplementary Table 2. Authorised phosphorus and sodium additives classified according to their phosphorus and sodium content, relative to the total molecular weight.

| Low content (<25% by weight) | | | | Medium content (25-39% by weight) | | | | High content (≥40% by weight) | | | |
| --- | --- | --- | --- | --- | --- | --- | --- | --- | --- | --- | --- |
| E-number | Name | Formula | Weight (%) | E-number | Name | Formula | Weight (%) | E-number | Name | Formula | Weight (%) |
| Phosphorus | | | |  | | | |  | | | |
| E 101 (ii) | Riboflavin-5’-phosphate | C_17_H_20_N_4_NaO_9_P | 6.4 | E 338 | Phosphoric acid | H_3_PO_4_ | 31.6 |  |  |  |  |
| E 339 (i) | Monosodium phosphate | Monohydrate: NaH_2_PO_4_ · H_2_O | 22.2 | E 339 (i) | Monosodium phosphate | Anhydrous:  NaH_2_PO_4_ | 25.6 |  |  |  |  |
| E 339 (i) | Monosodium phosphate | Dihydrate: NaH_2_PO_4_ · 2H_2_O | 19.7 | E 341 (i) | Monocalcium phosphate | Anhydrous: Ca(H_2_PO_4_)_2_ | 25.9 |  |  |  |  |
| E 339 (ii) | Disodium phosphate | Na_2_HPO_4_ | 21.8 | E 343 (i) | Monomagnesium phosphate | Mg(H_2_PO_4_)_2_ | 27.8 |  |  |  |  |
| E 339 (iii) | Trisodium phosphate | Na_3_PO_4_ | 18.9 | E 343 (ii) | Dimagnesium phosphate | MgHPO_4_ | 25.2 |  |  |  |  |
| E 340 (i) | Monopotassium phosphate | KH_2_PO_4_ | 22.8 | E 450 (i) | Disodium diphosphate | Na_2_H_2_P_2_O_7_ | 27.6 |  |  |  |  |
| E 340 (ii) | Dipotassium phosphate | K_2_HPO_4_ | 17.6 | E 450 (ii) | Trisodium diphosphate | Anhydrous:  Na_3_HP_2_O_7_ | 25.1 |  |  |  |  |
| E 340 (iii) | Tripotassium phosphate | K_3_PO_4_ | 14.6 | E 450 (vii) | Calcium dihydrogen diphosphate | CaH_2_P_2_O_7_ | 28.4 |  |  |  |  |
| E 341 (i) | Monocalcium phosphate | Monohydrate: Ca(H_2_PO_4_)_2_ · H_2_O | 24.3 | E 451 (i) | Pentasodium triphosphate | Na_5_O_10_P_3_ | 25.0 |  |  |  |  |
| E 341 (ii) | Dicalcium phosphate | Anhydrous: CaHPO_4_ | 22.3 | E 541 | Sodium aluminium phosphate, acidic | Anhydrous:  Na_3_Al_2_H_15_(PO_4_)_8_ | 27.6 |  |  |  |  |
| E 341 (ii) | Dicalcium phosphate | Dihydrate: CaHPO_4_ · 2H_2_O | 17.6 | E 541 | Sodium aluminium phosphate, acidic | Tetrahydrate: NaAl_3_H_14_(PO_4_)_8_ · 4H_2_O | 26.1 |  |  |  |  |
| E 341 (iii) | Tricalcium phosphate | Ca_3_(PO_4_)_2_ | 19.8 |  |  |  |  |  |  |  |  |
| E 450 (ii) | Trisodium diphosphate | Monohydrate: Na_3_HP_2_O_7_ · H_2_O | 23.4 |  |  |  |  |  |  |  |  |
| E 450 (iii) | Tetrasodium diphosphate | Anhydrous: Na_4_P_2_O_7_ | 22.8 |  |  |  |  |  |  |  |  |
| E 450 (iii) | Tetrasodium diphosphate | Decahydrate: Na_4_P_2_O_7_ · 10H_2_O | 13.7 |  |  |  |  |  |  |  |  |
| E 450 (v) | Tetrapotassium diphosphate | K_4_P_2_O_7_ | 18.4 |  |  |  |  |  |  |  |  |
| E 450 (vi) | Dicalcium diphosphate | Ca_2_P_2_O_7_ | 24.1 |  |  |  |  |  |  |  |  |
| E 451 (ii) | Pentapotassium triphosphate | K_5_O_10_P_3_ | 20.4 |  |  |  |  |  |  |  |  |
| E 626 | Guanylic acid | C_10_H_14_N_5_O_8_P | 8.4 |  |  |  |  |  |  |  |  |
| E 627 | Disodium guanylate | C_10_H_12_N_5_Na_2_O_8_P | 7.6 |  |  |  |  |  |  |  |  |
| E 628 | Dipotassium guanylate | C_10_H_12_K_2_N_5_O_8_P | 7.0 |  |  |  |  |  |  |  |  |
| E 629 | Calcium guanylate | C_10_H_12_CaN_5_O_8_P | 7.6 |  |  |  |  |  |  |  |  |
| E 630 | Inosinic acid | C_10_H_13_N_4_O_8_P | 8.8 |  |  |  |  |  |  |  |  |
| E 631 | Disodium inosinate | C_10_H_11_N_4_Na_2_O_8_P· H_2_O | 7.8 |  |  |  |  |  |  |  |  |
| E 632 | Dipotassium inosinate | C_10_H_11_K_2_N_4_O_8_P | 7.2 |  |  |  |  |  |  |  |  |
| E 633 | Calcium inosinate | C_10_H_11_CaN_4_O_8_P | 7.9 |  |  |  |  |  |  |  |  |
| Sodium | | | |  | | | |  | | | |
| E 101 (ii) | Riboflavin-5’-phosphate | C_17_H_20_N_4_NaO_9_P | 4.8 | E 221 | Sodium sulphite | Anhydrous: Na_2_SO_3_ | 35.9 | E 339 (iii) | Trisodium phosphate | Na_3_PO_4_ | 42.0 |
| E 102 | Tartrazine | C_16_H_9_N_4_Na_3_O_9_S_2_ | 11.5 | E 250 | Sodium nitrite | NaNO_2_ | 33.0 | E 500 (i) | Sodium carbonate | Na_2_CO_3_ | 43.4 |
| E 104 | Quinoline yellow | C_18_H_9_NNa_2_O_8_S_2_ | 9.6 | E 251 | Sodium nitrate | NaNO_3_ | 26.8 | E 524 | Sodium hydroxide | NaOH | 57.5 |
| E 110 | Sunset yellow FCF | C_16_H_10_N_2_Na_2_O_7_S_2_ | 10.0 | E 262 (i) | Sodium acetate | Anhydrous: C_2_H_3_NaO_2_ | 27.7 |  |  |  |  |
| E 122 | Azorubine, carmoisine | C_20_H_12_N_2_Na_2_O_7_S_2_ | 9.1 | E 331 (iii) | Trisodium citrate | Anhydrous: C_6_H_5_O_7_Na_3_ | 26.5 |  |  |  |  |
| E 123 | Amaranth | C_20_H_11_N_2_Na_3_O_10_S_3_ | 11.3 | E 339 (ii) | Disodium phosphate | Na_2_HPO_4_ | 32.4 |  |  |  |  |
| E 124 | Ponceau 4R, cochineal red A | C_20_H_11_N_2_Na_3_O_10_S_3_ | 11.4 | E 450 (ii) | Trisodium diphosphate | Anhydrous: Na_3_HP_2_O_7_ | 28.0 |  |  |  |  |
| E 127 | Erythrosine | C_20_H_6_I_4_Na_2_O_5_H_2_O | 5.1 | E 450 (ii) | Trisodium diphosphate | Monohydrate: Na_3_HP_2_O_7_ ·H_2_O | 26.1 |  |  |  |  |
| E 129 | Allura red AC | C_18_H_14_N_2_Na_2_O_8_S_2_ | 9.2 | E 450 (iii) | Tetrasodium diphosphate | Anhydrous: Na_4_P_2_O_7_ | 33.9 |  |  |  |  |
| E 131 | Patent blue V | C_27_H_31_N_2_O_7_S_2_Na | 3.6 | E 451 (i) | Pentasodium triphosphate | Na_5_O_10_P_3_ | 30.9 |  |  |  |  |
| E 132 | Indigotine, indigo carmine | C_16_H_8_N_2_Na_2_O_8_S_2_ | 8.9 | E 500 (ii) | Sodium hydrogen carbonate | NaHCO_3_ | 27.2 |  |  |  |  |
| E 133 | Brilliant blue FCF | C_37_H_34_N_2_Na_2_O_9_S_3_ | 5.7 | E 500 (iii) | Sodium sesquicarbonate | Na_2_CO_3_ · NaHCO_3_ · 2H_2_O | 30.4 |  |  |  |  |
| E 142 | Green S | C_27_H_25_N_2_NaO_7_S_2_ | 3.6 | E 514 (i) | Sodium sulphate | Anhydrous: Na_2_SO_4_ | 32.3 |  |  |  |  |
| E 151 | Brilliant black BN, black PN | C_28_H_17_N_5_Na_4_O_14_S_4_ | 10.5 |  |  |  |  |  |  |  |  |
| E 155 | Brown HT | C_27_H_18_N_4_Na_2_O_9_S_2_ | 7.0 |  |  |  |  |  |  |  |  |
| E 211 | Sodium benzoate | C_7_H_5_O_2_Na | 15.9 |  |  |  |  |  |  |  |  |
| E 215 | Sodium ethyl p-hydroxybenzoate | C_9_H_9_O_3_Na | 12.1 |  |  |  |  |  |  |  |  |
| E 219 | Sodium methyl p-hydroxybenzoate | C_8_H_7_O_3_Na | 13.0 |  |  |  |  |  |  |  |  |
| E 221 | Sodium sulphite | Heptahydrate:  Na_2_SO_3_7H_2_O | 17.9 |  |  |  |  |  |  |  |  |
| E 222 | Sodium bisulphite | NaHSO_3_ | 21.9 |  |  |  |  |  |  |  |  |
| E 223 | Sodium metabisulphite | Na_2_S_2_O_5_ | 23.9 |  |  |  |  |  |  |  |  |
| E 262 (i) | Sodium acetate | Trihydrate: C_2_H_3_NaO_2_ · 3H_2_O | 16.8 |  |  |  |  |  |  |  |  |
| E 262 (ii) | Sodium diacetate | C_4_H_7_NaO_4_ | 16.0 |  |  |  |  |  |  |  |  |
| E 281 | Sodium propionate | C_3_H_5_O_2_Na | 23.7 |  |  |  |  |  |  |  |  |
| E 285 | Sodium tetraborate (borax) | Na_2_B_4_O_7_ | 22.6 |  |  |  |  |  |  |  |  |
| E 301 | Sodium ascorbate | C_6_H_7_O_6_Na | 11.5 |  |  |  |  |  |  |  |  |
| E 316 | Sodium erythorbate | C_6_H_7_O_6_Na · H_2_O | 10.5 |  |  |  |  |  |  |  |  |
| E 325 | Sodium lactate | C_3_H_5_NaO_3_ | 20.3 |  |  |  |  |  |  |  |  |
| E 331 (i) | Monosodium citrate | Anhydrous: C_6_H_7_O_7_Na | 10.6 |  |  |  |  |  |  |  |  |
| E 331 (i) | Monosodium citrate | Monohydrate:  C_6_H_7_O_7_Na · H_2_O | 9.8 |  |  |  |  |  |  |  |  |
| E 331 (ii) | Disodium citrate | C_6_H_6_O_7_Na_2_ · 1.5H_2_O | 17.3 |  |  |  |  |  |  |  |  |
| E 331 (iii) | Trisodium citrate | Dihydrate:  C_6_H_5_O_7_Na_3_ · 2H_2_O | 23.2 |  |  |  |  |  |  |  |  |
| E 331 (iii) | Trisodium citrate | Pentahydrate: C_6_H_5_O_7_Na_3_ · 5H_2_O | 19.6 |  |  |  |  |  |  |  |  |
| E 335 (i) | Monosodium tartrate | C_4_H_5_O_6_Na · H_2_O | 11.7 |  |  |  |  |  |  |  |  |
| E 335 (ii) | Disodium tartrate | C_4_H_4_O_6_Na_2_ · 2H_2_O | 19.7 |  |  |  |  |  |  |  |  |
| E 337 | Potassium sodium tartrate | C_4_H_4_O_6_KNa · 4H_2_O | 8.1 |  |  |  |  |  |  |  |  |
| E 339 (i) | Monosodium phosphate | Anhydrous: NaH_2_PO_4_ | 19.0 |  |  |  |  |  |  |  |  |
| E 339 (i) | Monosodium phosphate | Monohydrate: NaH_2_PO_4_ · H_2_O | 16.6 |  |  |  |  |  |  |  |  |
| E 339 (i) | Monosodium phosphate | Dihydrate: NaH_2_PO_4_ · 2H_2_O | 14.6 |  |  |  |  |  |  |  |  |
| E 350 (i) | Sodium malate | Hemihydrate: C_4_H_4_Na_2_O_5_ ½ H_2_O | 24.1 |  |  |  |  |  |  |  |  |
| E 350 (i) | Sodium malate | Trihydrate: C_4_H_4_Na_2_O_5_ 3H_2_O | 19.4 |  |  |  |  |  |  |  |  |
| E 350 (ii) | Sodium hydrogen malate | C_4_H_5_NaO_5_ | 14.4 |  |  |  |  |  |  |  |  |
| E 356 | Sodium adipate | C_6_H_8_Na_2_O_4_ | 23.7 |  |  |  |  |  |  |  |  |
| E 385 | Calcium disodium ethylenediaminetetraacetate | C_10_H_12_O_8_CaN_2_Na_2_ · 2H_2_O | 11.0 |  |  |  |  |  |  |  |  |
| E 450 (i) | Disodium diphosphate | Na_2_H_2_P_2_O_7_ | 20.5 |  |  |  |  |  |  |  |  |
| E 450 (iii) | Tetrasodium diphosphate | Decahydrate: Na_4_P_2_O_7_ ·10H_2_O | 20.4 |  |  |  |  |  |  |  |  |
| E 514 (i) | Sodium sulphate | Decahydrate: Na_2_SO_4_ · 10H_2_O | 14.3 |  |  |  |  |  |  |  |  |
| E 514 (ii) | Sodium hydrogen sulphate | NaHSO_4_ | 18.6 |  |  |  |  |  |  |  |  |
| E 521 | Aluminium sodium sulphate | AlNa(SO_4_)_2_ | 9.4 |  |  |  |  |  |  |  |  |
| E 535 | Sodium ferrocyanide | Na_4_Fe(CN)_6_ · 10H_2_O | 18.9 |  |  |  |  |  |  |  |  |
| E 541 | Sodium aluminium phosphate, acidic | Anhydrous:  Na_3_Al_2_H_15_(PO_4_)_8_ | 7.7 |  |  |  |  |  |  |  |  |
| E 541 | Sodium aluminium phosphate, acidic | Tetrahydrate: NaAl_3_H_14_(PO_4_)_8_ · 4H_2_O | 2.4 |  |  |  |  |  |  |  |  |
| E 576 | Sodium gluconate | C_6_H_11_NaO_7_ | 10.5 |  |  |  |  |  |  |  |  |
| E 621 | Monosodium glutamate | C_5_H_8_NaNO_4_· H_2_O | 12.2 |  |  |  |  |  |  |  |  |
| E 627 | Disodium guanylate | C_10_H_12_N_5_Na_2_O_8_P | 11.3 |  |  |  |  |  |  |  |  |
| E 631 | Disodium inosinate | C_10_H_11_N_4_Na_2_O_8_P | 11.7 |  |  |  |  |  |  |  |  |
| E 640 (ii) | Sodium glycinate | C_2_H_5_NO_2_Na | 23.5 |  |  |  |  |  |  |  |  |
| E 952 (ii) | Sodium cyclamate | Anhydrous: C_6_H_12_NNaO_3_S | 11.3 |  |  |  |  |  |  |  |  |
| E 952 (ii) | Sodium cyclamate | Dihydrate: C_6_H_12_NNaO_3_S ·2H_2_O | 9.6 |  |  |  |  |  |  |  |  |
| E 954 (ii) | Sodium saccharin | C_7_H_4_NNaO_3_S·2H_2_O | 9.5 |  |  |  |  |  |  |  |  |

Chemical formula and molecular weight can vary for E 322, E 401, E 407, E 407a, E 415, E 418, E 440 (i), E 440 (ii), E 442, E 452 (i), E 452 (ii), E 452 (iii), E 452 (iv), E 466, E 468, E 469, E 470a, E 472c, E 481, E 554, E 634, E 635, E 1200, E 1404, E 1410, E 1412, E 1413, E 1414, E 1442, E 1450, and E 1451.
